# Development of a rock-bit interaction analytical model by considering the in-situ stresses for a bottom-hole element

**DOI:** 10.1038/s41598-024-56177-0

**Published:** 2024-03-11

**Authors:** Habiballah Zafarian, Mohammad Javad Ameri, Alireza Dolatyari

**Affiliations:** Department of Petroleum Engineering, Amirkabir University of Technology, Tehran, Iran

**Keywords:** Rock-bit interaction, Rock failure angle, Cutting force, Analytical model, PDC bit, Energy science and technology, Engineering, Mathematics and computing

## Abstract

PDC drill bits are an important part of drilling engineering, but improper selection or design can lead to decreased performance and increased costs. Then, accurate modeling of rock-bit interaction for Oil/gas well drilling is critical. Although several mathematical models are presented for this purpose, they have not been able to present a comprehensive model for the rock-bit interaction. In-situ stresses in real drilling conditions affect the force required for rock failure. However, the models proposed so far either have not considered the effects of in-situ stresses or have assumed that the rock failure angle in the downhole conditions is equal to the one calculated in the atmospheric conditions. In this work, after reviewing the background of studies conducted on the rock and bit interaction, with an analytical method, stresses applied to the bottom hole element are examined, including stresses resulting from bit and in-situ stresses. Based on the principle of superposition, the total stress imposed on the bottom hole element is calculated to determine the angle and force of rock cutting. Finally, a novel mathematical model of rock-bit interaction in vertical and deviated oil/gas wells drilling by Considering In-Situ Stresses is presented. Also, the study compares the current model to the Nishimatsu and Xin Ling models using data from a southwest field in Iran. The results show that the simplifying assumption made by previous models leads to a significant underestimation of the failure angle and the amount of force required to the rock failure, with reductions of up to 21% and 48%, respectively, in the case of a vertical well. In an inclined well, the current model predicts cutting force at about 0.14 of that predicted by the previous model.

## Introduction

The drill bit is the most fundamental and basic tool required for drilling operations, and it is responsible for crushing and cutting rock. Crushing induces fracturing and inelastic deformation in rocks, whereas cutting leads to the development of fractures and the formation of a large chip ^1^. While some researchers believe that rocks fail in a single mode, the acceptance of a condition where both mechanisms occur simultaneously is more widespread ^2,3^.

One of the most essential challenges that engineers encounter during drilling operations is selecting the optimal drill bit and its operating conditions. Drilling bits can be selected based on the conditions; however, for rotary drilling, they are classified into two broad categories: roller cone bits and fixed cutter bits, which each have different types. The PDC drill is a form of fixed cutter bit that has a key benefit over roller cone types in that it does not have a moving and sensitive component such as a bearing; therefore, the issue of its loss is substantially decreased. In the late 1970s, PDC drills first debuted in the oil industry.

The objective for introducing this innovative type of drill bit was to reduce drilling expenditures, which is considered a revolution in the oil industry. The issue, however, is not the price of the PDC drill bit but its impact on the drilling process and, as a result, the reduction of associated expenses. Because the development of cutting structure, dynamic stability, and steerability boosted the efficiency of drill bits in drilling operations. To determine the drill bit's drill-ability in relation to different formations, as well as its steerability and stability in various situations, we must first understand the types of main cutting forces and how to control them. To improve drilling operations and enhance ROP, we need to understand how a drill bit behaves, and the interaction between rock and the drill bit is critical in this respect.

Many studies have been conducted in this area to determine the best model for the interaction of rock and bit, and many models have been presented to examine this issue. All of these models investigated this problem by employing one of two experimental or analytical methodologies. In this paper, we will focus on analytical models.

Merchant ^4,5^ established the first analytical model of the cutting process for metal surface cutting. Rock cutting has been the subject of many analytical models, including Evans' theory of coal plowing that was introduced in 1962 ^3^. This theory suggests that the failure in the direction of the wedge tip is primarily caused by tensile forces, and it examines the cutting forces generated by a wedge-shaped tool when it penetrates rocks. In summary, Evans’ theory provides fundamental insights into the mechanics of rock cutting and has been widely studied in the field of mining and related industries. Evans’ model is not intended for cutting, but rather for the process of penetration.

In fact, assuming linear shear plane is not representative of real conditions, and to overcome this issue, critical tensile stresses along a path of fracture propagation were delineated to specify rock chipping. ^3,6,7^ This was predicated on the way that rocks behaved in uniaxial compression testing after they failed their tensile fusion; however, in tests involving rock cutting, powder-like chips were seen these models could not explain that. A more precise answer for estimating the force needed for rock cutting was given by Merchant’s model, which was further enhanced using the Mohr–Coulomb failure criterion ^8,9^.

Many studies in the area of rock cutting have been developed over time, to the point where one of the oldest and most powerful of them, given by Nishimatsu ^8^ in 1972, is one of the most important analytical models of rock-bit interaction. Shear forces are the underlying cause of brittle failure in Nishimatsu's model, while Roxborough and Philips created a cutting model for disk cutters ^10^. The rock cutting model introduced by Nakajima and Kinoshita in 1979 identifies crack propagation as the main mechanism of failure ^11^. Cheatham and Ho were the first to investigate the concept of rock-bit interaction in directional drilling in 1981.

They accomplished this by defining a linear relationship between the thrust force components and the drilling rate vector. The distinct contributions of rock anisotropy and bit anisotropy were clearly superior in their model ^1^. This model was further completed by Ho, and finally, in 1988, it led to a general linear relationship for the interaction of rock and bit, and thus, the law of rock-bit interactions should be able to describe the relationship between the forces and moments applied by the bit to the rock and express the depth and direction of penetration ^12^.

In 1992, Detournay and Defourny created a semi-empirical model based on the assumption of rock failure in two stages: first, frictional contact between the wear flat and rock interface; and second, rock removal, which formed the basis of subsequent studies. The rock removal in this model is proportional to the drilling depth, and the friction angle between the rock and the bit remains constant during the drilling ^2^. The results mentioned by Detournay and Defourny were criticized in many studies, while others, such as Dagrain ^13^ and Perneder ^14^, tried to confirm and improve them. Also, in 2007, Richard conducted research in this regard using a rock resistance device and presented a relationship between cutting force and cutting depth, which was based on two types of failure regimes, ductile and brittle. ^15^

In the ductile state, the cutting mechanism applied by the cutting tool is described as a flow of sheared rock ahead of the cutting surface. The rock is only cut to a small depth, it is in a so-called “plastic state,” it is not broken but is cut, and it is stated that in the case of a rectangular cutter, the cutting forces are proportional to the depth of penetration and there is a linear relationship between the cutting force and the cutting depth ^15^. A result that was proposed in 1978 by Cheatham ^1^ and also in 1996 by Adachi ^16^.

In brittle mode, also called chipping, cracks are created at the tip of the tool and propagate forward. When a crack reaches the surface of the sample, a chip is formed, and then it is separated by the cutter. In this case, the average force is not linearly related to the depth. Cutting force and cutting depth have an exponential connection, according to Richard ^15^. Most experimental investigations suggest a linear relationship between applied forces and cutting depth in rock.

In continuation of the analytical studies carried out regarding finding a relationship to describe the rock-bit interaction, Li and Itakura ^17^ in 2012, based on the model provided by Nishimatsu, were able to divide the effective processes in drilling into two processes: cutting and feeding. They provide a more complete comparison between the applied force of the bit and the parameters of the rock and bit, and based on this model, they present an analytical relationship to calculate the compressive uniaxial stress. But the issue that should be kept in mind is that the effect of in-situ stresses has not been taken into account in any of the stated models.

The study conducted by Demeng Che, Wu-Le Zhu, and Kornel F. Ehmann investigates the various mechanisms of rock cutting and how rock properties and cutting parameters influence the dominant mechanism. The authors’ experiments reveal that the chipping mechanism is more prevalent in brittle rocks with low compressive strength, while the crushing mechanism dominates in ductile rocks with high compressive strength. The study’s findings have important practical implications for designing rock cutting tools for different types of rocks and cutting conditions ^18^.

Xin Ling presented a model in 2019 that was a relatively more complete model to predict the failure force in PDC drill bits in the presence of in-situ stresses for a single cutter ^19^, continuing the most important actions taken in this field. Xin Ling and colleagues have developed a model to estimate rock force and failure angle, but it has a flaw.

Despite the fact that in-situ stresses are taken into account in this model, there are two issues with it. The first case is that during the calculation of the failure angle, the assumption of atmospheric conditions has been applied and the effect of in-situ stresses on the failure angle has been ignored, so this model predicts the same failure angles for different drilling depths if the rock type and back rake angle are constant, while this is not the case in reality. The second thing that should be revised and corrected in the presented model by Xin Ling is the rotation of in-situ stresses that were applied to the rock in the depth of the earth and rotation in order to find the force required to break the rock in the condition of the deviated well, which is due to the wrong assumption. This issue was handled incorrectly during the rotation of stress on the bottom-hole element of deviating well, resulting in a severe error.

In 2021, Chao Xiong and colleagues created an analytical model that incorporates both in-situ stress and induced thermal stress for a stringer PDC cutter. However, their study was limited to vertical wells, and the model’s complexity necessitated the use of finite element method to solve the solution ^20^.

In summary, several models as ^2,15,21,22^ have been introduced to predict force by correlating force data with cutting parameters through regression. These models, known as Phenomenological Models, can reasonably predict the required force within a specific range of parameters. However, they fall short in describing some inherent mechanisms of force response from a physical failure perspective. To overcome the limitations of these models, analytical models based on the complete stress distribution using fracture theory have been proposed ^7,23,24^. Given the complexities involved in these models, numerical computations are sometimes necessary for their solution ^25–27^. Thus, to simplify force models, the definition of critical stress distribution has been employed^3,8,9,28^. If constructed correctly, models of this category can provide an accurate representation of force along with a meaningful physical interpretation.

According to the discussed topics, the model provided by Xin Ling et al., is a more complete model in terms of considering the in-situ stresses compared to other presented models, but it does not consider the effect of the in-situ stresses on the failure angle as well as the incorrect rotation of the in-situ stresses applied to the to the rock element in the deviated well has caused errors.

In this article, by removing the simplifying assumption of the presented model by Xin Ling and correcting the rotation of in-situ stresses, an improved model has been presented that can be used in the design of drilling operations and the selection of a suitable drill bit, which can help to reduce the costs of drilling operations with a more accurate prediction.

## Rock-bit interaction modeling

In order to check the force required to break the rock, as well as the angle of the rock break and the parameters affecting them, all the stresses applied to the bottom-hole element of the well should be considered. In this section, the stresses caused by bit force are first investigated based on the single-cutter model and the model presented by Nishimatsu and Merchant. Then, the stresses on the bottom-hole element of the well, which are caused by the in-situ stresses, will be investigated. Finally, according to the principle of “superposition,” the stresses applied to the rock will be equal to the sum of the stresses created by the two mentioned parts. It should also be noted that the model is examined in the condition of a deviated well, and the condition of a vertical well is considered a special case of a well with $0^\circ$ deviation. It should also be noted that the model is considered in two-dimensional conditions, i.e., anisotropic horizontal stresses condition.

There are two main points to consider. Firstly, the force from the cutter is not affected by the well angle, as drilling is always done in a vertical position with the bit axis in line with the well axis and perpendicular to the well bottom. Secondly, the in-situ stresses must be rotated to match the well's degree of inclination in order to determine the stress applied to the bottomhole element. It is important to note that rotatable stresses are consistent across all elements, whereas variable stresses are dependent on the element. For instance, the stress applied perpendicularly to the bottomhole element is always equal to the pressure in the upper fluid column, regardless of the well inclination angle. Conversely, the stress applied to the right side of the bottomhole element is a combination of horizontal and vertical stress, which varies with the well inclination angle. This stress rotation has been problematic in previous studies, including that of Xin Ling.

### Methodology

In this study, the Nishimatsu model has been considered as the foundational model. By utilizing the rotation of in-situ stresses for deviated wells and also considering the angle of failure, the stresses resulting from in-situ stresses were determined. It is worth mentioning that the vertical stress on the bottom hole element is the weight of the fluid column. Furthermore, the stresses applied from the force imposed by the serpentine on the fracture plane were identified. Subsequently, based on the principle of superposition, the normal and shear stresses imposed on the rock element were determined. Using the Mohr–Coulomb failure criterion, the relationship between the force imposed by the serpentine, in-situ stresses, as well as the angle of failure and rock properties such as internal friction angle and rock strength, was elucidated.

To determine the angle of failure, it was assumed that the force exerted on the rock must be at its extremum. Using differentiation, the angle of rock failure was determined based on other parameters. The main difference between the current model and the one proposed by Jean Ling is that atmospheric conditions were assumed for calculating the angle of failure in Ling’s model, neglecting in-situ stresses. Additionally, the rotation of in-situ stresses was not properly accounted for. To validate the derived relationships, setting the principal stresses and well inclination equal to zero results in the equation provided by Nishimatsu. Therefore, the modeling has been done correctly. Furthermore, to evaluate the proposed model, a case study has been considered, where calculations have been performed at various depths and angles. These calculations were conducted in Microsoft Excel, and given the recursive relationship between force and failure angle in deviated wells, a trial-and-error method was employed. Subsequently, a more detailed examination of the model's expansion is undertaken.

### Stresses caused by cutting force

In this section, we will consider Nishimatsu's model as the basis of our calculations, and based on their assumptions, we will investigate and model the stress caused by the applied bit force.

**Figure 1:**
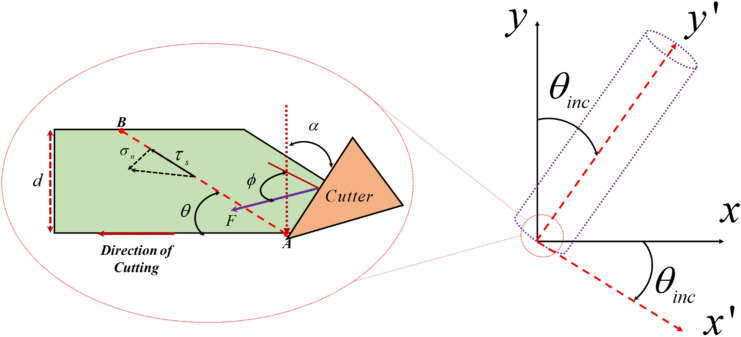
A schematic form of resultant forces analysis of the rock.

In Fig. 1, a schematic of the relevant well, which is deviated $\theta_{inc}$ from the vertical state, as well as the bottom hole element of the well and the forces and stresses applied from the single cutter side, are shown.

Let $AB$ be the rock fracture line at an angle $\theta$ to the plane perpendicular to the well axis, and consider $\lambda$ as the distance from point $A$ in the direction of the rock fracture line. The width of the fracture surface that is inside the plane is equal to $w$, and the depth of the rock fracture is equal to $d$. Basically, the line $AB$ is very small, so the rate of change of the resulting stress, which we represent with $P$, is very intense, and thus we consider it as a power function depending on the position relative to the point $A$, that is, $\lambda$. The total length of line $AB$ is equal to $d/\sin \theta$. Given that the maximum value of this stress is equal to $P_{0}$ at point $A$ and zero at point $B$, we can write the value of $P$ as a power function of $n$, the value of which is given by Nishimatsu for cement mortar and sandy tuff samples. This coefficient is related to the parameters of the drill tooth and is a function of back rake angle in the form of $n = 11.3 - 0.18\alpha$.We can obtain the applied force, denoted by $F$, by integrating $P$ from point $A$ to point $B$.

In this way, $P_{0}$ can be reached with a displacement in the equation of $F$, and as a result, the relationship written for $P$ can be rewritten as a function of $F$ and $\lambda$. Considering that the value of stress $P$ is maximum at the beginning of the path $AB$, i.e. at $\lambda = 0$, by putting it in the equation, the value of $P$ at point $A$ is calculated. And by visualizing it on the fracture plane, which has an angle of $\theta$ with respect to the plane of the bottom of the well, we will reach the relations for the normal and shear stresses applied on the $AB$ line, which are in the form of relations (1) and (2), where $\tau_{s0}$, $\sigma_{n0}$ are, respectively, the shear and normal stresses applied to the rock in the rock failure plane.

1 $$ \sigma_{n0} = \frac{n + 1}{{dw}}F \cdot \sin \theta \cdot \sin \left( {\theta + \phi - \alpha } \right) $$

2 $$ \tau_{s0} = \frac{n + 1}{{dw}}F \cdot \sin \theta \cdot \cos \left( {\theta + \phi - \alpha } \right) $$

In this way, we determined the stresses caused by the bit force. Now we can get to the stresses on the element caused by the in-situ stresses.

### The stresses on the bottom hole element caused by the in-situ stresses

Three types of stresses are applied to the rock element: minimum horizontal stress, maximum horizontal stress, and vertical stress. The effect of in-situ stresses at the location of the rock element is equivalent to the distant points, meaning that the stress applied to the rock is equal to the minimum and maximum horizontal stresses. This yields the stress resulting from the aforementioned stresses on the rock element. Also, the vertical stress applied to the rock is equal to the pressure caused by the hydrostatic column in the well. Due to the fact that the well has been rotated by the amount of $\theta_{inc}$ relative to the horizon, we must first transfer the in-situ stresses to the horizontal plane of the well, which is perpendicular to the axis of the well, which is done with the help of the transfer matrix presented in Eq. (3).

3 $$ R = \left[ {\begin{array}{*{20}c} {\cos \theta_{inc} } & {\sin \theta_{inc} } & 0 \\ { - \sin \theta_{inc} } & {\cos \theta_{inc} } & 0 \\ 0 & 0 & 1 \\ \end{array} } \right] $$

Therefore, if the in-situ stress tensor of the area on the rock element is defined in the form of Eq. (4), with its rotation to the plane perpendicular to the axis of the well, with the help of operation $R\sigma_{p} R^{t}$, the effect of the in-situ stresses in the desired plane can be calculated. It should be noted that the vertical stress applied to the bottom hole element is always the pressure of the fluid column, so instead of $\sigma_{yy} = \sigma_{v} \cos^{2} \theta_{inc} + \sigma_{h} \sin^{2} \theta_{inc}$, we use the pressure of the fluid column ($\rho gh$). Therefore, the stress matrix in the plane perpendicular to the well axis will be in the form of relation (5).

4 $$ \sigma_{p} = \left[ {\begin{array}{*{20}c} {\sigma_{h} } & 0 & 0 \\ 0 & {\sigma_{v} } & 0 \\ 0 & 0 & {\sigma_{H} } \\ \end{array} } \right] $$

5 $$ \sigma_{f} = \left[ {\begin{array}{*{20}c} {\sigma_{xx} } & {\tau_{xy} } & {\tau_{xz} } \\ {\tau_{yx} } & {\sigma_{yy} } & {\tau_{yz} } \\ {\tau_{zx} } & {\tau_{zy} } & {\sigma_{zz} } \\ \end{array} } \right] = \left[ {\begin{array}{*{20}c} {\sigma_{v} \sin^{2} \theta_{inc} + \sigma_{h} \cos^{2} \theta_{inc} } & {\frac{{\sigma_{v} - \sigma_{h} }}{2}\sin 2\theta_{inc} } & 0 \\ {\frac{{\sigma_{v} - \sigma_{h} }}{2}\sin 2\theta_{inc} } & {\rho gh} & 0 \\ 0 & 0 & {\sigma_{h} } \\ \end{array} } \right] $$

t the stresses calculated in Eq. (5) are the stress value in the plane perpendicular to the well axis, while we should consider the stresses calculated in the plane of failure. As a result, we must again transfer the stresses obtained in relation (5) with the rotation of $\frac{\pi }{2} - \theta$ to the desired fracture plane using the appropriate transfer matrix and operation $\sigma_{on \;failure \;Plane} = R_{f} \sigma_{f} R_{f}^{t}$, and we have calculated the stresses on the bottom hole element caused by the in-situ stresses on the failure plane. The idea of superposition may now be utilized to determine the total stress applied to the bottom hole element, which is generated by in-situ stresses and the cutting force.

### The total stress on the rock element at the bottom hole and calculation of the angle and force of rock failure

This allows us to compute the total stresses imparted to the rock element at the bottom hole using the superposition method. Ultimately, the rock element in the fracture plane at the bottom of the deviated well will experience shear and vertical stresses, which will equalize the values in Eqs. (6) and (7), respectively. The influence of pore pressure on the applied stresses, which is represented by P and b is Biot’s Number, is taken into consideration since it should be remembered that the needed stresses must be achieved efficiently to be included in the intended failure criterion.

6 $$ \sigma_{n} = \left( {\sigma_{v} \sin^{2} \theta_{inc} + \sigma_{h} \cos^{2} \theta_{inc} } \right)\sin^{2} \theta + \sigma_{3} \cos^{2} \theta - bP + \frac{n + 1}{{dw}}F \cdot \sin \theta \cdot \sin \left( {\theta + \phi - \alpha } \right) $$

7 $$ \begin{aligned} \tau_{s} & = - \frac{1}{2}\left( {\sigma_{3} - \sigma_{v} \sin^{2} \theta_{inc} - \sigma_{h} \cos^{2} \theta_{inc} } \right) \cdot \sin \left( {2\theta } \right) + \frac{1}{2}\left( {\sigma_{v} - \sigma_{h} } \right)\sin \left( {2\theta_{inc} } \right) \cdot \cos \left( {2\theta } \right) \\ & \;\;\;\; + \frac{n + 1}{{dw}}F \cdot \sin \theta \cdot \cos \left( {\theta + \phi - \alpha } \right) \\ \end{aligned} $$

According to Bellin et al.^29^, the failure of rocks and similar brittle materials can be predicted by analyzing the Mohr stress circle envelope. This envelope often takes the form of a straight line in the compressive normal stress region for many types of rocks. Other studies, including those conducted by Seliami et al.^30^ and Kühne ^31^, have shown that the Mohr–Coulomb criterion is a useful method for explaining changes in cutting force resulting from rock strength. As long as the maximum stress on the fracture line meets the failure criterion, the rock will break.

Now, according to the applied stresses on the rock element, we must check the rock failure angle with the help of a suitable failure criterion. Due to the fact that the rock failure regime is ductile, the suitable criterion for rock failure in this condition is the Mohr–Coulomb criterion, which is in the form of $\left| \tau \right| = S_{s} + \sigma_{n} \tan k$, where $S_{s}$ is shear strength and $k$ is the internal friction angle. The force required to the rock failure as a function of $\theta$ can be calculated by plugging the obtained stresses into the relationship related to the Mohr–Coulomb failure criterion. To find the minimum force required to break the rock and the angle of rock failure, we differentiate the force with respect to $\theta$ and then perform mathematical operations to arrive at relations (8) to (10) for the minimum force required to break the rock and the angle of rock failure. Note that by setting $\left( {\theta_{inc} = 0} \right)$ equal to zero, we will reach the desired relationship for the vertical well.

8 $$ \tan \left( {2\theta } \right) = \frac{{\frac{n + 1}{{dw}}F_{b} .\cos \left( {k + \phi - \alpha } \right) + \left( {\sigma_{x} - \sigma_{y} } \right)\cos k - 2\tau_{xy} \sin k}}{{\frac{n + 1}{{dw}}F_{b} .\sin \left( {k + \phi - \alpha } \right) + \left( {\sigma_{x} - \sigma_{y} } \right)\sin k - 2\tau_{xy} \cos k}} $$

9 $$ \begin{aligned} F_{b} & = \left( {\sigma_{y} - \sigma_{x} } \right)\left[ {2\tan \theta \cdot \cos k + \left( {1 - \tan^{2} \theta } \right)\sin k} \right] + \left( {1 + \tan^{2} \theta } \right)\left[ {2S_{s} \cdot \cos k + \left( {\sigma_{x} + \sigma_{y} - P} \right)\sin k} \right] \\ & \;\;\;\; + 2\tau_{xy} \left[ {2\sin k \cdot \tan \theta - \left( {1 - \tan^{2} \theta } \right)\cos k} \right] \\ \end{aligned} $$

10 $$ \left[ {\begin{array}{*{20}c} {\sigma_{x} } \\ {\sigma_{y} } \\ {\tau_{xy} } \\ \end{array} } \right] = \left[ {\begin{array}{*{20}c} {\sigma_{v} \sin^{2} \theta_{inc} + \sigma_{h} \cos^{2} \theta_{inc} } \\ {\sigma_{3} } \\ {\frac{{\sigma_{v} - \sigma_{h} }}{2}\sin 2\theta_{inc} } \\ \end{array} } \right] $$

## Results and discussion

**Figure 2:**
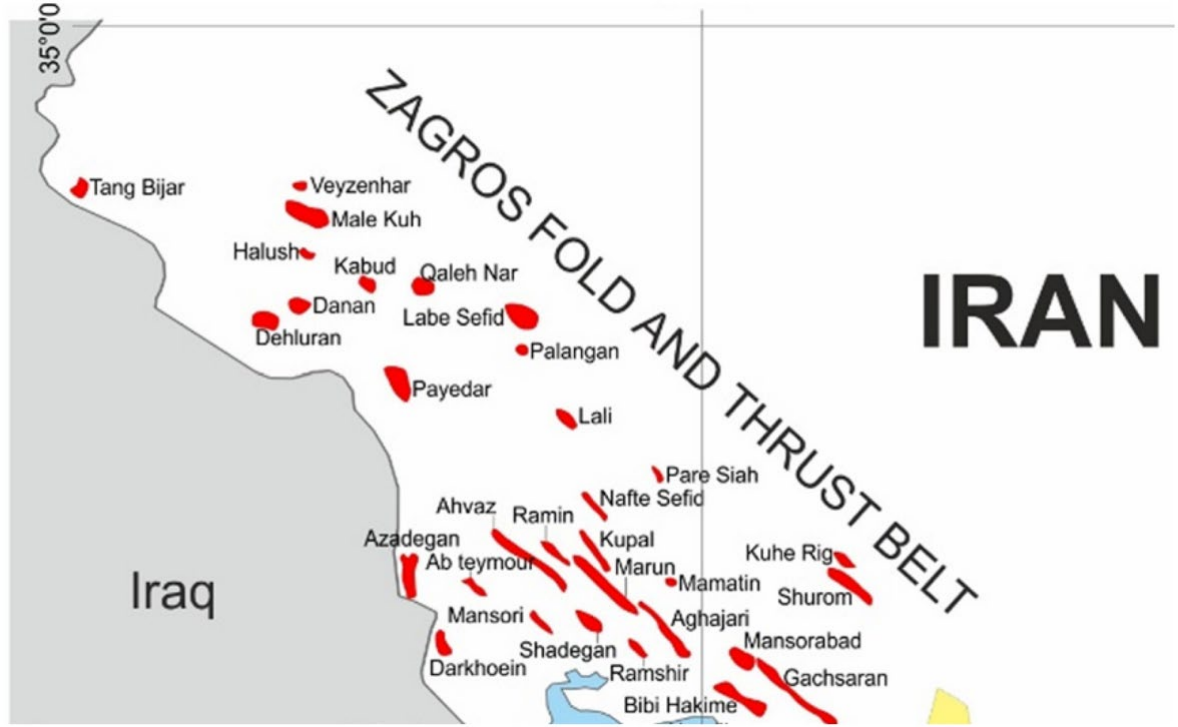
Location of the case study’s region.

**Figure 3:**
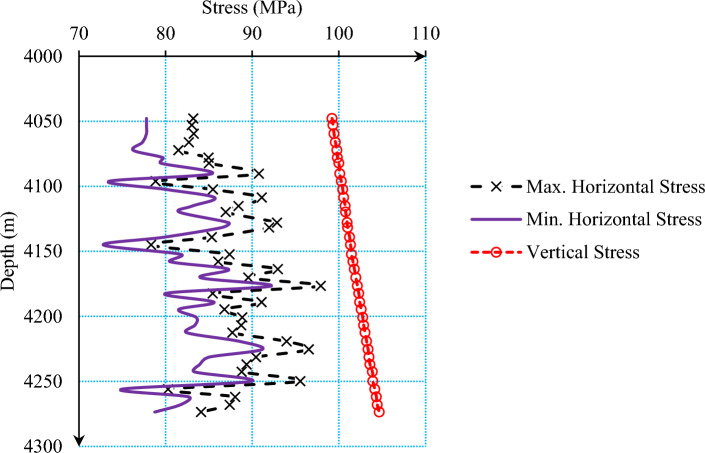
Changes in the in-situ stresses of the studied reservoir versus depth.

In this part, in order to examine the presented model by Xin Ling and the model presented by Nishimatsu, a case study of wells drilled in Iran's oil fields is considered. This case study is located in one of the fields in the southwest of Iran, which has 4212 million barrels of oil in place. Figure 2 In Table 1, data about the mechanical properties of the desired reservoir are presented. The changes in the in-situ stresses compared to the depth in the case study obtained with the help of MEM are presented in Fig. 3.

**Table 1:** Mechanical properties of case study.

Pore pressure [MPa]	Poisson’s ratio [–]	Biot’s coefficient [–]	Internal friction angle [°]	Dynamic elasticity modul [GPa]	Static elasticity modul [GPa]	UCS [MPa]
47.0	0.302	0.7	44.96	41.49	10.37	47.035

### Examining changes in rock failure angles

As stated, Nishimatsu did not consider the in-situ stresses in his model, and Xin Ling, despite considering the effect of in-situ stresses derived from the obtained equation for force before entering it into the equations, has ignored the effect of in-situ stresses. Thus, the equation of both of them was in the form of relation (11) or in the general form of $\theta_{Nish} = \theta_{Xin} = \frac{\pi }{4} - \frac{1}{2}\left( {k - \alpha + \phi } \right)$, but in the present model it is in the form of relation (8), which has a reciprocal relationship with relation (9).

11 $$ \theta_{Nish} = \theta_{Xin} = \frac{2m + 1}{4}\pi - \frac{k - \alpha + \phi }{2};\;\;\;m = 0,1,2, \ldots $$

As it is known, if the influence of stresses caused by in-situ stresses and rock pore pressure is not considered, we will arrive at the Nishimatsu’s model and the model provided by Xin Ling for the failure angle. It should also be noted that according to the presented model, the rock failure angle is a function of the mechanical properties of the rock, the angle between the rock and bit, and the applied in-situ stresses.

We will compare the results of the presented model with the stated conditions for the case study in order to compare the results of the presented model with the results of two other models. It should be noted that $d = 0.002\left[ m \right]$ and $w = 0.003 \left[ m \right]$ are considered in this investigation, and the well is in vertical conditions $\left( {\theta_{inc} = 0} \right)$.

**Figure 4:**
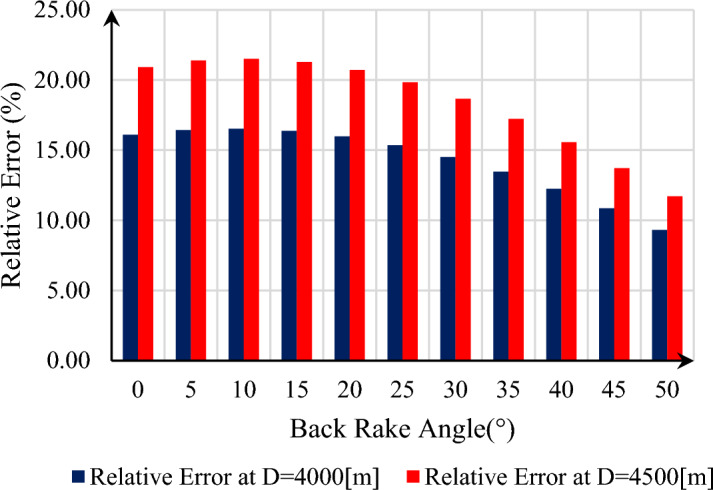
Relative error of failure angle versus back rake angle of Xin Ling model in comparison with present model.

If we consider the ratio of this difference to the value calculated by the presented model as a relative error, the relative error in the desired depth is between 9.31 and 16.52%, which increases as the back rake angle increases up to 10°, then decreases to $\alpha = 50^\circ$. This difference grows as the depth is increased to 4500 m, due to an increase in the in-situ stresses, because the failure angle of the rock in the presented model by Xin Ling does not change with depth change, whereas in the model presented in this research, depth change affects the failure angle. In this way, this relative error can be seen graphically in Fig. 4. As is known, the relative error rate is 11.7–21.51 percent.

**Figure 5:**
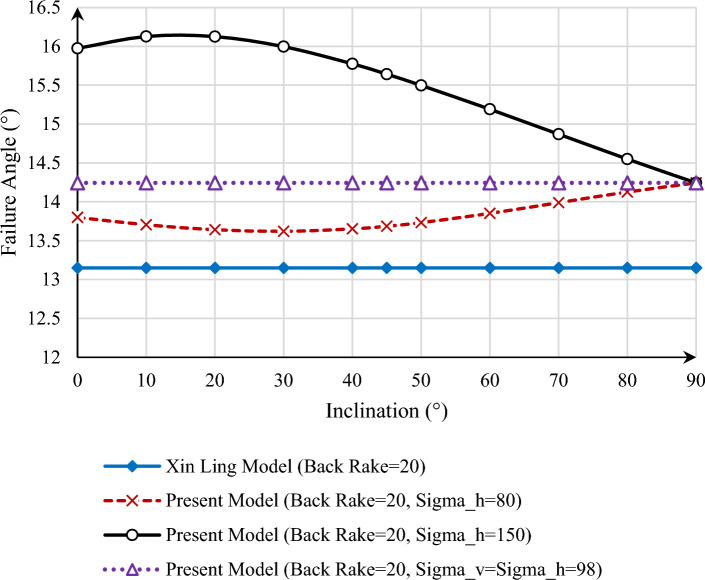
Failure angle versus inclination of borehole for back rake angle = 20, in Xin Ling and present model for different in-situ stresses.

Another point that is considered in this research is the effect of the well inclination on the rock failure angle, which was not considered in the previous models. As a result, we anticipate that the failure angle of the rock in the model provided by Xin Ling will remain constant as the well angle changes. By examining this issue at a depth of 4000 m and changing the angle of deviation of the well between 0, which is the vertical condition, and 90, which corresponds to the condition of the horizontal well, the two presented models can be drawn in the condition of $\alpha = 20^\circ$ in Fig. 5. As can be seen, the current model’s trend of changes is such that when $\sigma_{h} < \sigma_{v}$, i.e., $\sigma_{h} = 80\;\left[ {{\text{MPa}}} \right]$, increasing the well deviation angle up to 30° reduces the difference between the failure angle in the presented model and the model provided by Xin Ling, and, once again, this issue follows an increasing trend from 30° on. This issue is due to the difference between $\sigma_{h}$ and $\sigma_{v}$ values.

As can be seen, for the condition that $\sigma_{h} > \sigma_{v}$, that is, $\sigma_{h} = 150 \;\left[ {{\text{MPa}}} \right]$, the value of the rock failure angle increases up to the well with a 15° inclination and then decreases from 15° to 90°. It should be noted that this amount of deviation depends on the difference between $\sigma_{h}$ and $\sigma_{v}$ and how one compensates the other in the existing relationship between these two stresses in the presented relations.

If we consider the condition where $\sigma_{H} = \sigma_{h} = \sigma_{v}$, the failure angle of the rock will not change for the presented model by Xin Ling and the current model compared to the changes in the well inclination angle and provide a constant value, and this issue can be justified according to the presented relationships because, in the model provided by Xin Ling, the well inclination does not affect the failure angle. In the presented model, by equalizing two vertical and horizontal stresses, $\tau_{xy} = 0$ and $\sigma_{x} = \sigma_{v} = \sigma_{h}$, the effect of the well deviation angle on the rock failure angle has been removed. These conditions are also shown in Fig. 5 for $\alpha =20^\circ$, which shows that the failure angle of the rock is constant and does not change with the changes in the angle of deviation of the well from the vertical to the horizon.

**Figure 6:**
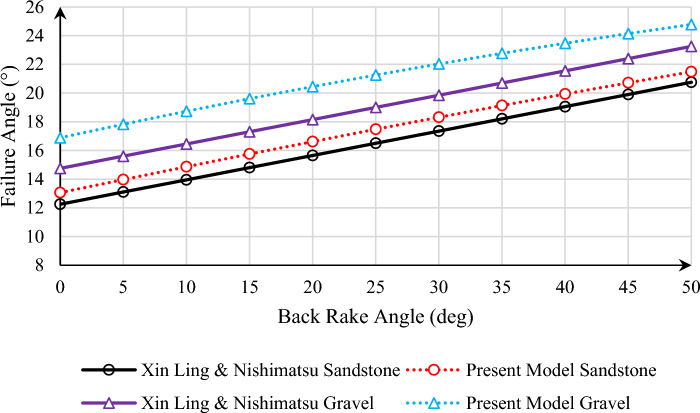
Failure angle versus back rake angle for different models for sandstone and gravel rock types.

Now we can check the effect of the type of rock in the desired model. In this part, two types of sandstone and gravel are considered, whose shear strength values are equal to 10 MPa and 600 kPa, and whose internal friction angle values are 40° and 35°, respectively. In this way, the amount of rock failure angle for the two mentioned cases is presented in the present model and the model that provided by Xin Ling in Fig. 6 for the vertical well. As it is shown, in the model provided by Xin Ling, by changing from sandstone to gravel, the failure angle has increased by 2.5°, which is due to the direct effect of half of the internal friction angle, while in the presented model in this article, the effect of the rock parameters is different. As a result of this increase in the value of the internal friction angle, the value of the rock failure angle has not been constant and varies from 3.29° to 3.82°. As shown in the graph, with the increase in shear strength and internal friction angle, the difference between the two models has decreased.

### The force required to rock cutting

The presented relation by Nishimatsu is in the form of relation (12). As it is clear from the stated relationship, from the rock properties, only the rock’s shear strength and internal friction angle are effective in the force equation related to the Nishimatsu’s model, and there is no effect of in-situ stresses. If, in the relation presented in this article, the in-situ stresses as well as the pore pressure are considered equal to zero and the conditions of the well are considered equal to the vertical, we will reach the relation presented by Nishimatsu.

12 $$ F = \frac{{2dS_{s} }}{n + 1}\frac{\cos k}{{1 - \sin \left( {k + \phi - \alpha } \right)}} $$

The relationship presented by Xin Ling is in the form of Eq. (13), where the values of $R_{1} ,R_{2} ,R_{3}$ are presented in relationships (14)–(16).

13 $$ F = R_{1} + R_{2} + R_{3} $$

14 $$ R_{1} = \frac{{\sigma_{1} \left[ {1 + \cos \left( {2\alpha_{c} + k + \theta + \phi } \right)} \right]}}{{\sin \left( {\phi + \theta } \right) - \sin k - \cos \left( {\theta + \phi } \right)\sin \left( {k + \phi + \theta } \right)}} $$

15 $$ R_{2} = \frac{{2S_{s} .\cos k}}{{\sin \left( {\theta + \phi } \right) - \sin k - \cos \left( {\theta + \phi } \right)\sin \left( {\theta + \phi + k} \right)}}\frac{{\sin \left( {\frac{1}{2}\left( {\theta + \phi - k} \right)} \right)}}{{\left( {n + 1} \right)\cos \left( {\frac{1}{2}\left( {\theta + \phi + k} \right)} \right)}} $$

16 $$ R_{3} = \frac{{\sigma_{3} \left[ {1 - \cos \left( {2\alpha_{c} + k + \theta + \phi } \right)} \right]}}{{\sin \left( {\theta + \phi } \right) - \sin k - \cos \left( {\theta + \phi } \right)\sin \left( {\theta + \phi + k} \right)}} $$

It should be noted that in the current model and the Nishimatsu model, the inclination of the well is considered equal to $\alpha_{c}$, as is the value of $\theta = \frac{\pi }{2} - \alpha$, where $\alpha$ is the back rake angle. By placing the above items, the relationships related to the model provided by Xin Ling can be expressed in the format presented in this article.

As mentioned, Nishimatsu calculated the force required to break the rock in the absence of in-situ stresses, and Xin Ling also caused an error with the wrong rotation of stress on the bottom hole element of the deviated well. One of the most obvious mistakes is the influence of the inclination angle of the well on the pressure of the fluid column. If the well is vertical or horizontal, the pressure of the fluid column does not change, and the pressure of the fluid column on the bottom hole is always equal to $\rho gh$. Considering this and the fact that the $\sigma_{v}$ effect does not exist in the model provided by Xin Ling, the results should show a significant difference.

**Figure 7:**
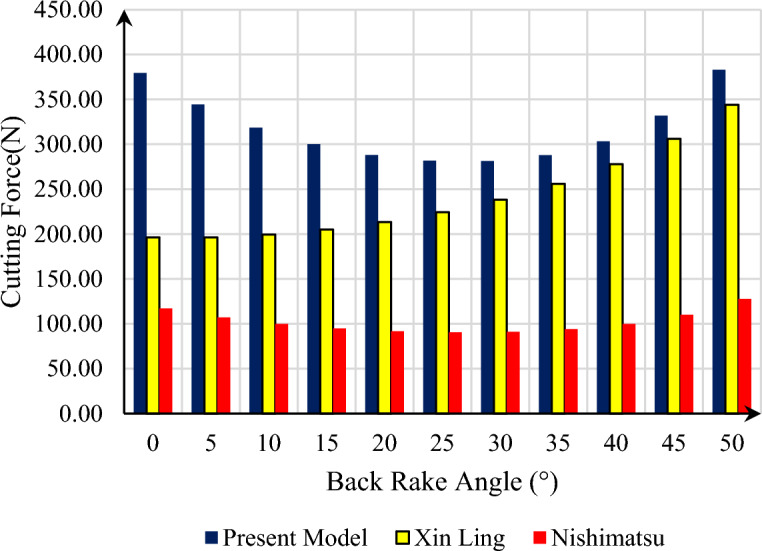
Comparison between calculated cutting force for present model, presented model by Xin Ling et al., and Nishimatsu in different back rake angle.

In order to investigate the force required to break the rock, the values of $w = 0.003\;\left[ {\text{m}} \right]$ and $d = 0.002\;\left[ {\text{m}} \right]$ have been considered, and this problem has been investigated at a depth of 4000 m. This issue is presented for the three models presented in Fig. 7, and as can be seen in the presented model, the force required to break the rock is significantly higher at back rake angles smaller than 20° compared to the presented model by Xin Ling. If we consider their relative difference compared to the model provided by Xin Ling, this relative difference changes between 7.4 and 89.2%, which decreases with the increase in back rake angle. Also, as can be seen, both the Nishimatsu model and the current model have a similar trend, which is more tangible in the current model. And the detection of the back rake angle, the angle at which failure occurs with the lowest possible force, is clearer and is determined at an angle of 25°. But in the model provided by Xin Ling, this trend is an upward trend, which makes it difficult to determine the right angle for the design of the drill bit.

If we consider the effect of the well rotation on the back rake angle equal to 20° in the deviated well, then it can be seen that the incorrect effect of the well deviation in the model provided by Xin Ling has caused a sudden increase in the force required to break the rock with the increase of the well's angle, but in the current model, with the change in the inclination angle of the well, this increase in the cutting force that is required to break the rock does not occur suddenly.

**Figure 8:**
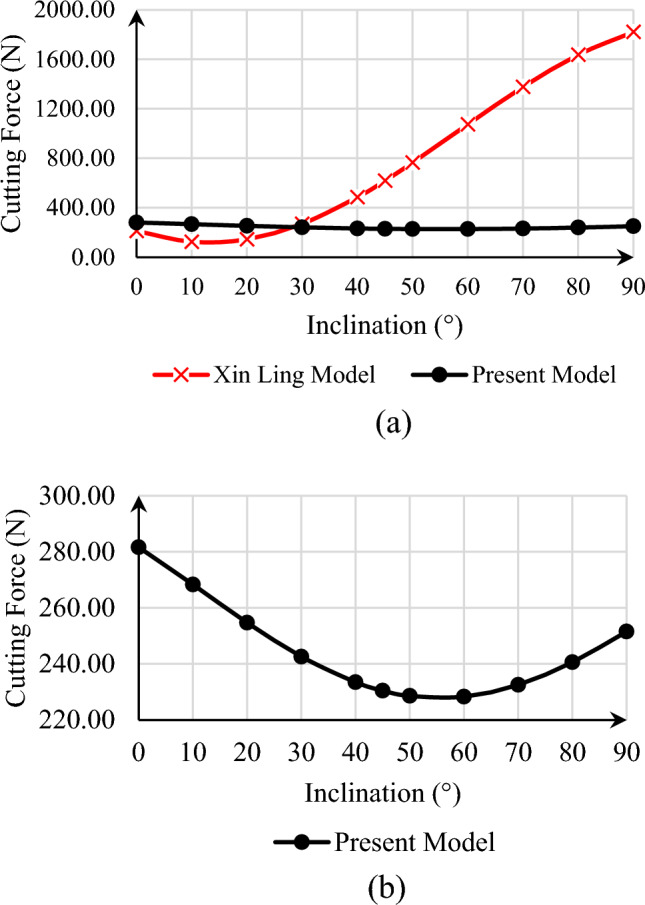
Calculated cutting force comparison of present model and presented model by Xin Ling et al., for back rake angle = 20° in different borehole inclination.

This issue is fully shown in Fig. 8. As can be seen, with the increase in the inclination of the well, the force required to break the rock first follows a decreasing trend, and then it increases, which is reasonable considering the change in vertical stress from the pressure of the fluid column to the stress caused by the weight of the overburdened rock, but this increase in the model presented by Xin Ling is such that the reduction of the cutting force that is required to break the rock eventually occurs up to the well inclination of about 10° and then the increasing trend becomes so intense that the order of the numbers changes sharply. This causes the applied force to the cutting tool to be overestimated according to the cutting force which specified by Xin Ling and, as a result, cause damage to the drill bit during the drilling operation or when considering the necessary measures for the drill bit's design, greatly increasing drilling costs. As it is clear in Part a of Fig. 8, the impact of changes in the inclination angle of the well on the amount of force required to the rock failure in the same conditions as the model provided by Xin Ling suddenly changes drastically, which the reason was stated.

But in the presented equation in the current model, according to the effects of in-situ stresses and the stated relationships, it can be clearly seen that with the increase in the inclination of the well, the effect of overburden stress increases, As a result, in the examined case, the force required to break the rock goes through a decreasing trend up to an angle of 60°, and from then on, it has an increasing trend. It should be noted that despite the increase in cutting force from about 60° onward, the force increase is not as intense as the presented model by Xin Ling. The changes related to the model presented in this article can be seen more clearly in Part b of Fig. 8. It should be noted that the Nishimatsu model is not considered because it does not account for deviation from well conditions and the presence of in-situ stresses.

**Figure 9:**
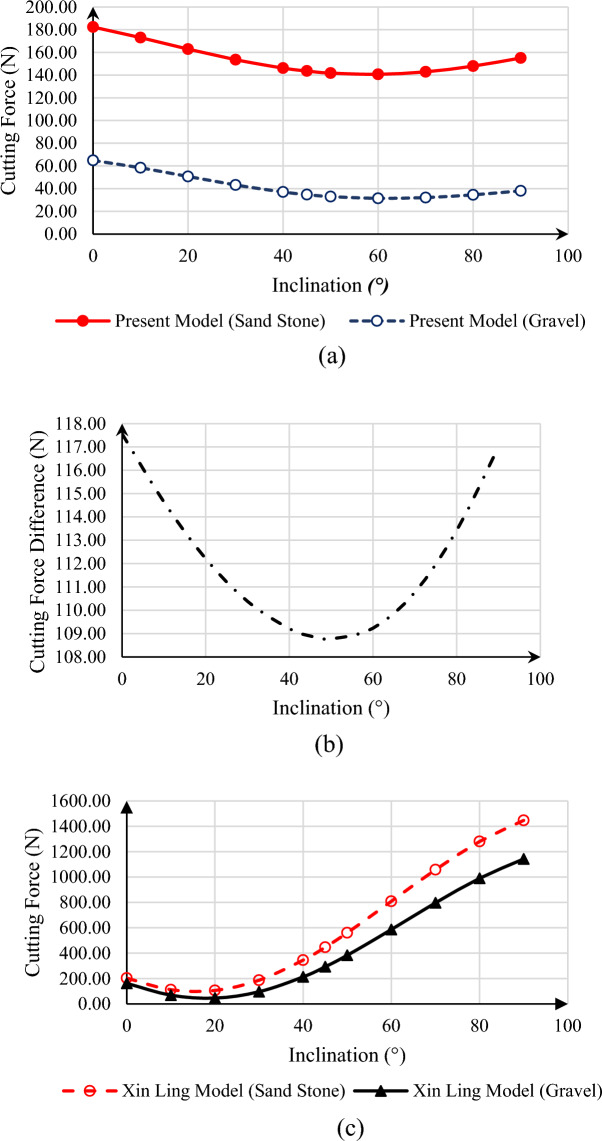
Calculated cutting force for present model and presented model by Xin Ling et al. for different inclination with comparing impact of rock type (**a**) cutting force versus well inclination in back rake angle = 20° for present model for sandstone and gravel, (**b**) sandstone and gravel cutting force difference versus well inclination in back rake angle = 20° for present model, (**c**) cutting force versus well inclination in back rake angle = 20° for the model provided by Xin Ling for sandstone and gravel.

Another issue that is being investigated is the effect of the rock type, which will affect the properties of the rock. We’ll check this again for two cases of sandstone and gravel. Thus, the results are presented in Fig. 9.

The shear strength and internal friction angle decrease as one moves from sandstone to gravel, as shown in Fig. 8. The force needed for cutting through the rock has also generally decreased, but this decreasing trend is not constant in relation to the well inclination; the figure also shows the difference between the two. It reaches its minimum value at an angle of 50°. It should be noted that the curves of both the mentioned rock models have a decreasing trend up to an angle of 60° of well deviation from the vertical state, and from 60° on, they follow an increasing trend, which is due to the fact that the applied stresses are the same as the conditions presented in Fig. 8. Furthermore, the model with the well inclination angle changes forecasts fewer changes than the one supplied by Xin Ling, suggesting that the latter model underestimated the rock failure force. Figure 8’s section (c) makes it evident that the rock-cutting force changes in the Xin Ling model are significantly greater than those indicated by the present model.

## Conclusion

Accurately modeling rock-bit interaction is critical. Over time, various analytical models have been developed, with Nishimatsu's model being the first and most well-known. However, previous models have had a primary limitation in that they all assumed atmospheric conditions when examining rock failure, whereas underground drilling operations occur in non-atmospheric conditions. The in-situ stresses and mud column pressure in the well have a significant impact on the force required to break the rock, and thus, it is important to account for these factors when modeling rock-bit interaction. In order to design and select a suitable bit for drilling operation better estimation of rock failure angle and required cutting force is needed.

So, an analytical model to investigate the interaction between rock and bit has been developed in this article, taking into account the conditions of the deviated well as well as the presence of in-situ stresses. This action has been carried out by considering the effect of in-situ stresses on rock failure angle and the effect of in-situ stresses in the directional well, and as a result, an effective analytical relationship has been developed to calculate the force and rock failure angle in the conditions of drilling operations. The comparison of the present model with previous models, which were analyzed using information from one of Iran’s oil reservoirs, indicates an improvement in the results obtained with the new model. The most important results of this study can be mentioned as follows:

1. As mentioned earlier, the Nishimatsu model has been adopted as the basis for the current model. The Nishimatsu model determines the failure angle under atmospheric conditions and does not consider in-situ stresses. Additionally, the Nishimatsu model does not apply to deviated wells. The latest model developed based on the Nishimatsu model, considering in-situ stresses and wellbore inclination, is the Jean Ling model. Despite considering in-situ stresses, the Xin Ling model determines the rock failure angle before incorporating in-situ stresses, thus presenting the failure angle under atmospheric conditions. Furthermore, the rotation related to the wellbore inclination is not properly accounted for in the Xin Ling model. Therefore, these aspects have been corrected in the proposed model. The rock failure angle is calculated by considering in-situ stresses, and the effect of wellbore inclination is properly taken into account. This adjustment has led to improved results.
2. Taking into account the effect of in-situ stresses on rock failure angle, the simplifying assumption of equality of rock failure angle in atmospheric conditions and bottom hole conditions was removed, and an improvement was made in this part. Based on the studied reservoir information, the current model can improve the results of failure angle at a depth of 4500 m in the vertical well condition more than 20% compared to the results of other models.
3. In addition to the mechanical characteristics of the rock and overburden, the rock failure angle is a function of the rock’s internal friction angle, shear strength, angle between the rock and bit, geomechanical parameters (pore pressure, horizontal stress, and hydrostatic column of the drilling fluid in the well), and the inclination angle of the well.
4. Rock failure angle and rock cutting force should both be functions of the drilling depth and the angle of the bit tooth. As we go deeper, the in-situ stresses increase, resulting in a larger error in the estimates for the rock failure angle and rock cutting force when we utilize other methods. Therefore, the use of other methods such as Xin Ling at low depths (low in-situ stresses) will not lead to a large difference between the results of the model and reality, but with the increase in depth, this difference increases, and it is suggested to use the new model presented to calculate the force.
5. In the present model, the effect of the well inclination angle on the failure angle and the force required for rock failure were investigated and the results showed a strong difference between the present model and the model provided by Xin Ling in the conditions of the deviated well, which is due to the incorrect rotation of the in-situ stresses in the presented model by Xin Ling and also the effect of the rotation on the hydrostatic pressure column of the fluid. For example, at an angle of 90°, which corresponds to a horizontal well situation, for a back rake angle of 20°, the present model predicts a cutting force equal to 0.14 of the model provided by Xin Ling.
6. Although the difference in failure force calculations between the present model and other models decreases with increasing tooth angle, the difference in conventional rock and bit angles is relatively large. For one example, at a depth of 4500 m, as the back rake angle increases, the relative error of the failure angle decreases from 20.92% for a back rake of 0° to 11.7% for a back rake angle of 50°, but in the back rake angle range of about 10°,–20°, the relative error is about 21.5%. The same thing is true about the rock cutting force, and on average, 31% relative error is observed.

## Data Availability

All data generated or analyzed during this study are included in this published article.

## Abbreviations

$\alpha$: Back rake angle
$\phi$: Angle between direction of resultant force $F$ and the normal of front edge of PDC cutter
$\theta$: Angle between the fracture line and horizontal plane
$d$: Depth of cut
$F$: Resultant forces on rocks
$$\theta_{inc}$$: Well inclination
$P_{L}$: Resulting stress at point A
$\tau_{s0}$: The shear stresses applied to the rock in the rock failure plane
$\sigma_{n0}$: Normal stress applied to the rock in the rock failure plane
$\sigma_{v}$: Vertical principal stress (overburden)
$\sigma_{h}$: Horizontal principal stress
$$S_{s}$$: Shear strength
$k$: Internal friction angle
